# Deubiquitinase USP17 Regulates Osteoblast Differentiation by Increasing Osterix Protein Stability

**DOI:** 10.3390/ijms242015257

**Published:** 2023-10-17

**Authors:** Myeong Ji Kim, Meiyu Piao, Yuankuan Li, Sung Ho Lee, Kwang Youl Lee

**Affiliations:** Research Institute of Pharmaceutical Sciences, College of Pharmacy, Chonnam National University, Gwangju 61186, Republic of Korea; audwl0815@gmail.com (M.J.K.); my101park@gmail.com (M.P.); lyk208943@jnu.ac.kr (Y.L.)

**Keywords:** USP17, deubiquitinase, Osterix, osteoblast differentiation

## Abstract

Deubiquitinases (DUBs) are essential for bone remodeling by regulating the differentiation of osteoblast and osteoclast. USP17 encodes for a deubiquitinating enzyme, specifically known as ubiquitin-specific protease 17, which plays a critical role in regulating protein stability and cellular signaling pathways. However, the role of USP17 during osteoblast differentiation has not been elusive. In this study, we initially investigated whether USP17 could regulate the differentiation of osteoblasts. Moreover, *USP17* overexpression experiments were conducted to assess the impact on osteoblast differentiation induced by bone morphogenetic protein 4 (BMP4). The positive effect was confirmed through alkaline phosphatase (ALP) expression and activity studies since ALP is a representative marker of osteoblast differentiation. To confirm this effect, *Usp17* knockdown was performed, and its impact on BMP4-induced osteoblast differentiation was examined. As expected, knockdown of *Usp17* led to the suppression of both ALP expression and activity. Mechanistically, it was observed that USP17 interacted with Osterix (Osx), which is a key transcription factor involved in osteoblast differentiation. Furthermore, overexpression of *USP17* led to an increase in Osx protein levels. Thus, to investigate whether this effect was due to the intrinsic function of USP17 in deubiquitination, protein stabilization experiments and ubiquitination analysis were conducted. An increase in Osx protein levels was attributed to an enhancement in protein stabilization via USP17-mediated deubiquitination. In conclusion, USP17 participates in the deubiquitination of Osx, contributing to its protein stabilization, and ultimately promoting the differentiation of osteoblasts.

## 1. Introduction

Bone remodeling plays a crucial role in the adaption of the skeleton to its strength requirements for responding to physical stresses and maintaining mineral homeostasis [1,2]. Moreover, this process entails dynamic and intricate interactions between bone resorption and formation [3]. Thus, understanding the mechanisms and regulation of bone remodeling holds significant implications in physiological contexts, such as bone development and pathological conditions, including aging, fracture healing, and osteoporosis [4,5]. The last decades have witnessed a great progression in the treatment of bone disorders, including osteoporosis. For example, the traditional anti-resorptive agents, represented by bisphosphonates, have been predominantly employed for osteoporosis through inhibiting bone resorption [6]. As anti-resorptive agents are unable to efficiently restore bone mass and quality, recent strategies that promote both bone formation and resorption processes to maintain and enhance bone density have received considerable interest [7,8]. Therefore, it is important that a study be conducted to determine the genes or chemicals that regulate osteoblast differentiation and elucidate their mechanism of action.

Osterix (Osx), also known as Sp7 (specificity protein 7), is a key transcription factor that plays a critical role in bone development and skeletal homeostasis [9]. It is a member of the Sp/KLF (specificity protein/Krüppel-like factor) family of transcription factors and is primarily expressed in osteoblasts, which are the cells responsible for bone formation [10]. It regulates the synthesis and mineralization of the bone matrix by promoting the expression of *collagen type I*, *alkaline phosphatase* (*ALP*), *osteocalcin* (*OC*), and other osteoblast-specific genes [11,12]. Additionally, Osx collaborates with other transcription factors, such as Runx2, to orchestrate the sequential activation of osteoblast-specific genes, promoting the maturation and functionality of osteoblasts [13]. Osx-deficient mice display severe defects in skeletal development, which are characterized by impaired osteoblast differentiation, reduced bone formation, and skeletal deformities [14]. Understanding the role of Osx in bone development and homeostasis has clinical implications for skeletal disorders and bone-related diseases. Thus, therapeutic strategies targeting Osx and its downstream signaling pathways may offer potential approaches for bone regeneration, fracture healing, and the treatment of skeletal disorders.

USP17 encodes a deubiquitinating enzyme, specifically known as ubiquitin-specific protease 17, which plays a critical role in the regulation of protein stability and cellular signaling pathways [15,16]. USP17 encodes for a protein consisting of several domains, including a ubiquitin-specific protease domain and a ubiquitin-associated (UBA) domain, which are crucial for its enzymatic activity and protein–protein interactions [17]. USP17 has been implicated in various cellular processes, including protein degradation [18], DNA damage repair [19], and cell cycle regulation [20]. Emerging evidence suggests that the dysregulation of either USP17 expression or activity may contribute to the development and progression of several diseases [21], thereby highlighting its potential as a therapeutic target or diagnostic biomarker in these conditions. However, the functions of USP17 in bone devolvement and diseases remain unknown.

In this study, we aimed to elucidate the role of USP17 in osteoblast differentiation and its underlying mechanism. To examine the effect of USP17 in osteoblast differentiation, we utilized a system, which involved the treatment of pre-myocyte C2C12 cells with bone morphogenetic protein 4 (BMP4) to induce osteoblast differentiation. We found that USP17 promotes osteoblast differentiation by enhancing the stabilization of Osx protein through deubiquitination, which is mediated by its enzymatic activity.

## 2. Results

### 2.1. USP17 Promotes Osteoblast Differentiation in BMP4-Induced C2C12 Cells

**Figure 1:**
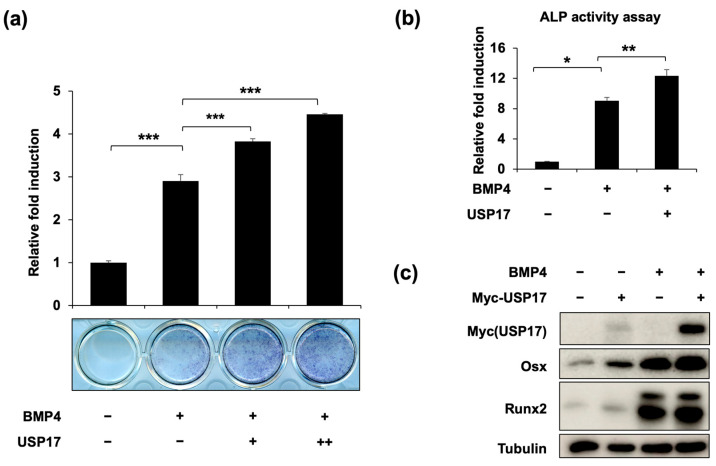
USP17 promotes osteoblast differentiation in BMP4-induced C2C12 cells. C2C12 cells were transfected with empty vector or Myc-USP17 (0.25, 0.5 μg) and treated with or without BMP4 for 72 h. (**a**) The presentative image and quantification of ALP staining. (**b**) The ALP activity was measured using a commercial kit. The data are presented as the mean ± SEM. * *p* < 0.05, ** *p* < 0.01, *** *p* < 0.001 compared to BMP4-treated group. (**c**) The protein levels of Myc-USP17, Osx, Runx2, and tubulin were measured by IB. Tubulin was used as a loading control.

**Figure 2:**
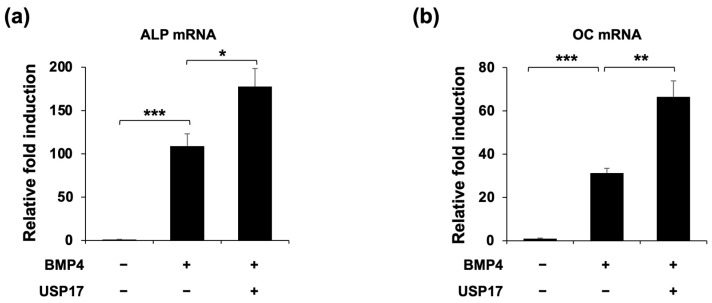
USP17 increases osteogenic gene expression in BMP4-induced C2C12 cells. C2C12 cells were transfected with empty vector or Myc-USP17 and treated with or without BMP4 for 48 h. (**a**,**b**) The mRNA levels of *ALP* and *OC* were measured by RT-qPCR and normalized against GAPDH. The data are presented as the mean ± SEM. * *p* < 0.05, ** *p* < 0.01, *** *p* < 0.001 compared to BMP4-treated group.

To investigate whether USP17 could regulate the differentiation of osteoblasts, we conducted *USP17* overexpression experiments to assess the impact of USP17 on ALP expression, a marker of early osteoblast differentiation [22]. Upon *USP17* overexpression, both the expression (Figure 1a) and activity (Figure 1b) of ALP were increased. Additionally, we examined the expression of key transcription factors, Runx2 and Osx, which are involved in osteoblast differentiation. We observed an increase in the expression of Runx2 and Osx in the BMP4-treated group and specifically noted a significant upregulation of Osx upon *USP17* overexpression (Figure 1c). Furthermore, the expression of *ALP* and *OC*, factors associated with osteoblast differentiation, were both increased upon *USP17* overexpression (Figure 2). In summary, these findings suggest that USP17 plays a role in the positive regulation of osteoblast differentiation.

### 2.2. Usp17 Knockdown Suppresses Osteoblast Differentiation in BMP4-Induced C2C12 Cells

**Figure 3:**
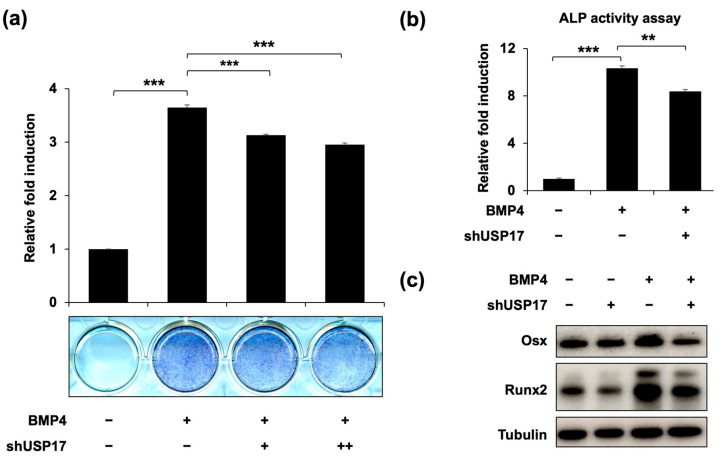
*Usp17* knockdown suppresses osteoblast differentiation in BMP4-induced C2C12 cells. C2C12 cells were transfected with pSuper retro puro empty vector or sh*Usp*17 plasmid (0.25, 0.5 μg) and treated with or without BMP4 for 72 h. (**a**) The presentative image and quantification of ALP staining. (**b**) The ALP activity was measured using a commercial kit. The data are presented as the mean ± SEM. ** *p* < 0.01, *** *p* < 0.001 compared to the BMP4-treated group. (**c**) The protein levels of Osx, Runx2, and tubulin were measured by IB. Tubulin was used as a loading control.

**Figure 4:**
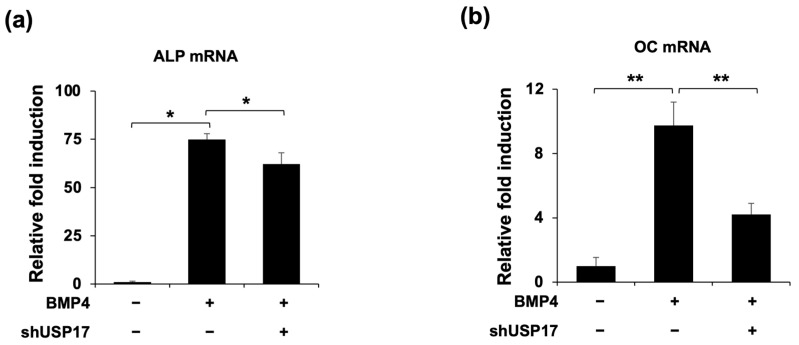
*Usp17* knockdown decreases osteogenic gene expression in BMP4-induced C2C12 cells. C2C12 cells were transfected with pSuper retro puro empty vector or sh*Usp*17 plasmid and treated with or without BMP4 for 48 h. (**a**,**b**) The mRNA levels of *ALP* and *OC* were measured by RT-qPCR and normalized against GAPDH. The data are presented as the mean ± SEM. * *p* < 0.05, ** *p* < 0.01 compared to the BMP4-treated group.

To confirm the previous findings, we examined the effects of *Usp17* knockdown on osteoblast differentiation. It was confirmed that the increased ALP expression and activity, induced by BMP4 treatment, were suppressed by *Usp17* knockdown (Figure 3a,b). Moreover, the protein expressions of Runx2 and Osx were inhibited by *Usp17* knockdown (Figure 3c). Additionally, the mRNA levels of *ALP* and *OC*, which are the genes associated with osteoblast differentiation, were upregulated by BMP4 treatment, and suppressed by *Usp17* knockdown (Figure 4). Overall, *Usp17* knockdown inhibited BMP4-induced osteoblast differentiation in C2C12 cells.

### 2.3. USP17 Interacts with Osx

**Figure 5:**
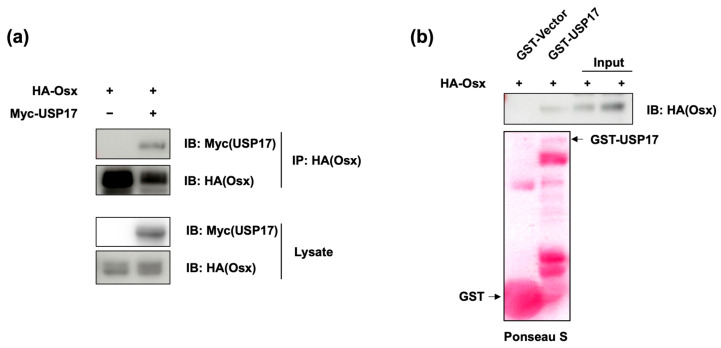
USP17 interacts with Osx. HEK 293 cells were transfected with HA-Osx or co-transfected with Myc-USP17. (**a**) The interaction between Osx and USP17 was examined by IP. (**b**) The interaction between purified USP17 and Osx was detected by GST pull-down assay and detected by IB. Expression of GST proteins was identified by Ponceau S staining (GST-vector: first line, GST-USP17: second line).

To investigate the underlying mechanism of how USP17 affects osteoblast differentiation, we performed experiments to explore the relationship between USP17 and the Osx protein, while considering various possibilities. To examine the interaction between these two proteins, IP (immunoprecipitation) and GST-pull down assays were performed. As shown in Figure 5a, it was confirmed that an interaction occurred between USP17 and Osx. Furthermore, it was verified that these two proteins bound directly to each other (Figure 5b). These results suggest that the effects of USP17 on osteoblast differentiation are potentially mediated through the Osx protein.

### 2.4. USP17 Increases Osx Protein Expression

**Figure 6:**
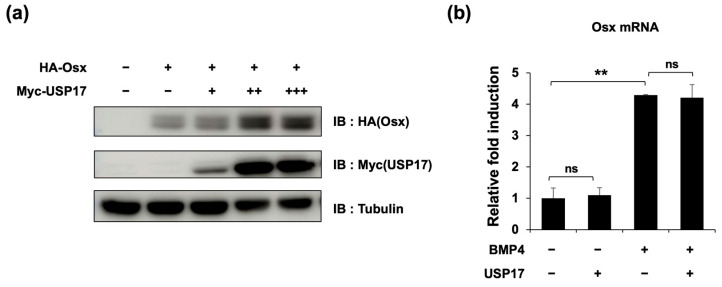
USP17 increases Osx protein expression. (**a**) HEK 293 cells were transfected with HA-Osx and increasing amounts of Myc-USP17 (0.125, 0.25, 0.5 μg). The protein levels were detected by IB. (**b**) C2C12 cells were transfected with empty vector or USP17 and treated with or without BMP4 for 48 h. The mRNA level of *Osx* was measured by RT-qPCR. The data are presented as the mean ± SEM. ns, not significant. ** *p* < 0.01 compared to the control group.

To determine the relationship between the previously observed interaction and its impact on Osx expression, we examined Osx expression at various concentrations of *USP17* overexpression. The results showed a concentration-dependent increase in the Osx protein levels (Figure 6a). However, it was confirmed that *USP17* overexpression, regardless of the presence of BMP4, did not affect *Osx* mRNA levels (Figure 6b). These data suggest that USP17 positively regulates Osx protein levels without affecting its transcription.

### 2.5. USP17 Increases Osx Protein Stability by Suppressing Its Polyubiquitination

**Figure 7:**
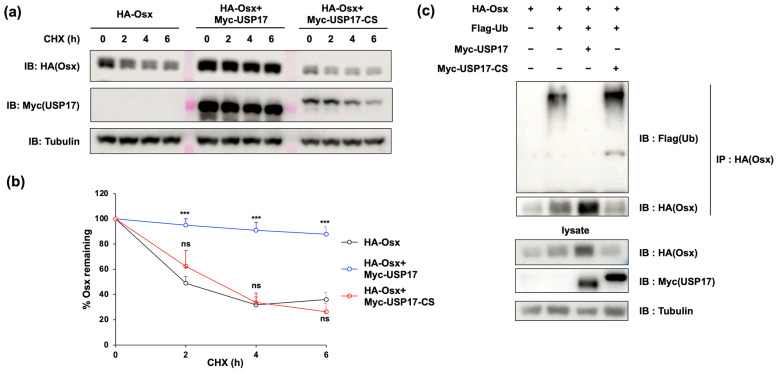
USP17 increases Osx protein stability by suppressing Osx ubiquitination. (**a**,**b**) HEK 293 cells were transfected with HA-Osx, Myc-USP17, and Myc-USP17-CS. After transfection, the cells were treated with CHX (40 μg/mL) for the indicated time. The protein levels of Osx, Myc-USP17, and tubulin were detected by IB. The intensities of protein (Osx) were quantified by Image J Version 1.52k. ns, not significant. *** *p* < 0.001 compared to the control group. (**c**) HEK 293 cells were transfected with HA-Osx, Flag-Ub, Myc-USP17, and Myc-USP17-CS. The ubiquitination of Osx was detected by IP and measured by IB.

Given that USP17 affects protein expression without altering its transcription, this suggests a mechanism related to protein stability, which can be mediated by the well-known role of USP17 in deubiquitination. Initially, protein stability was assessed using a CHX chase assay and revealed a significant increase in Osx stability upon *USP17* overexpression (Figure 7a,b). However, the overexpression of a mutant C89S (CS) targeting the enzymatic activity site of USP17 did not impact Osx stability (Figure 7a,b). Subsequently, the deubiquitination effect of USP17 was examined. As expected, overexpression of the *USP17* led to a substantial reduction in the expression of polyubiquitinated Osx (Figure 7c). In contrast, overexpression of USP17-CS did not show the effect previously observed with USP17 (Figure 7c). Collectively, the enzymatic activity of USP17 appears to facilitate the deubiquitination of Osx protein, resulting in enhanced stability and abundance of Osx protein.

## 3. Discussion

As previously mentioned, the balance between bone-forming osteoblasts and bone-resorbing osteoclasts is crucial in bone remodeling [23]. Disruption in this balance can lead to bone disorders, such as osteoporosis [24], and osteogenesis imperfecta [25]. Recently, the ubiquitin-proteasome system, which is a key component of post-translational modifications, has been reported to play a pivotal role in bone remodeling [26]. Interestingly, the administration of the proteasome inhibitor bortezomib was found to enhance mesenchymal stem cell osteogenic differentiation through the regulation of Runx2 [27]. Additionally, bortezomib stimulated bone formation by inhibiting the degradation of the key components, including β-catenin, Dkk1, and Gli3, in the Wnt/β-catenin signaling pathway, which is crucial in the differentiation of osteoblasts [28]. Furthermore, bortezomib was found to inhibit the activation of the RANKL-mediated NF-κB signaling pathway in osteoclasts [29]. Ixazomib, another proteasome inhibitor, promoted osteoblast formation and activity of osteoprogenitor cells from multiple myeloma patients through the activation of TCF/β-catenin signaling, unfolded protein response via IRE1α–XBP1 pathway, and upregulation of BMP2 in mesenchymal stromal cell (MSC) precursors [30,31]. Furthermore, Ixazomib activates the Sonic Hedgehog (SHH) pathway by inducing the nuclear translocation of Gli1 in human MSCs and osteoblast differentiation [30]. However, it is essential to identify other key players involved in E3 ligases and DUBs, to overcome the side effects resulting from off-target effects associated with shutting down the entire proteasome system.

Ubiquitin-specific proteases (USPs) play a crucial role in the regulation of signal transduction pathways during osteoblastogenesis [26,32]. BMPs and transforming growth factor β (TGFβ) are key factors in osteogenesis, particularly BMP-2, -4, and -7, which are known for their pro-osteogenic functions [33]. These BMPs regulate the induction of osteoblast-specific markers, such as ALP and OC, through the canonical BMP signaling pathway, which involves SMAD 1/5/8 phosphorylation [34]. USPs, notably USP4, USP11, and USP15, have emerged as critical regulators in bone function. Mechanically, USP4 influences the TGFβ/BMP pathway by deubiquitinating the TGFβ1 receptor, thereby stabilizing TGFβ signaling [35]. It also modulates the non-canonical TGFβ pathway and regulates Wnt/β-catenin signaling in osteoblasts [36]. USP11 targets the effectors of canonical TGFβ signaling, such as SMAD7 and ALK5 [37], while USP15 enhances TGFβ and BMP responses and regulates Wnt/β-catenin signaling [38]. In the context of mesenchymal commitment and differentiation, several USPs, including USP7 and USP34, are known to play pivotal roles in osteogenic differentiation by controlling the stability and activity of key regulators [39,40]. Furthermore, USP53 is implicated in the anabolic action of parathyroid hormones in osteoblasts [41]. In summary, USPs are integral in the modulation of signal transduction pathways, which are crucial for osteoblast function and bone formation, thereby highlighting their significance in bone biology and suggesting avenues for future research into their intricate roles and interactions. Notably, there have been no reported roles for USP17 in the process of bone remodeling. Instead, it is reported that USP17 is upregulated in osteosarcoma and promotes cell proliferation, metastasis, and epithelial–mesenchymal transition by stabilizing SMAD4 [42]. This report suggests the potential involvement of the *USP17* gene in bone remodeling, particularly in osteoblast differentiation.

USPs not only control osteogenic differentiation and bone formation but also regulate osteoclast differentiation and function. For example, although USP7 is known to regulate osteoclast differentiation, there is still ongoing debate. One group reported that USP7 acts as a positive regulator for osteoclast differentiation through its interaction with high-mobility group protein 1 (HMGB1) [43], while another group proposed that USP7 serves as a negative regulator for osteoclast differentiation via dual effects of attenuating tumor necrosis factor receptor-associated factor 6 (TRAF6)/TAK1 Axis and Stimulating stimulator of interferon gene (STING) Signaling [44]. Meanwhile, USP15 cooperates with charged multivesicular body protein 5 (CHMP5) to stabilize IκBα, resulting in decreased receptor activator of NF-κB ligand (RANKL)-mediated NF-κB activation and osteoclast differentiation [45]. USP18 acts as a negative regulator by deconjugating ISGylation, and its deficiency leads to heightened RANKL-mediated osteoclastogenesis, resulting in an osteopenic phenotype both in vivo and in vitro [46,47]. USP34 acts as a negative regulator of osteoclastogenesis through RANK/RANKL-induced NF-κB signaling by deubiquitinating and stabilizing IκBα [48]. *Usp53* deficient osteoblasts and bone marrow adipocytes exhibited increased expression of Rankl by enhancing the interaction between the vitamin D receptor (VDR) and SMAD3. This leads to perturbation of bone remodeling and augmentation of osteoblast-dependent osteoclastogenesis [49]. Interestingly, it has been revealed that some USPs play a dual role in regulating both osteoblast and osteoclast differentiation. Specifically, the previously mentioned USPs—USP7, USP15, USP34, and USP53—fall into this category, hinting at the potential roles of USP17 in osteoclast differentiation and function. Therefore, further investigation into the impact of USP17 on the process of osteoclast differentiation and its expression and role in osteoporosis patients or animal models appears necessary.

Osx is recognized as an unstable protein, whose stability undergoes regulation through the ubiquitin-proteasome system [50]. Osx plays a pivotal role in osteoblast differentiation and is influenced by various post-translational modifications, including phosphorylation [51,52], methylation [53,54], and ubiquitination [55]. Phosphorylation of Osx by kinases, such as p38, ERK, and Akt modifies its transcriptional activities, while the phosphorylation of Ser76 and Ser80 residues in Osx is speculated as being critical for its interaction with Pin1 [56]. Ubiquitination of Osx by E3 ubiquitin ligase Cbl-b and c-Cbl enhances its degradation, when Cbl-b and c-Cbl negative regulate osteoblast differentiation [57]. Furthermore, proteasome inhibitors, such as bortezomib and ixazomib, have been shown to elevate Osx protein levels, a key transcription factor in osteoblasts, thereby indicating the significance of proteasomal degradation in the regulation of osteogenic regulators [58]. This study delves into the molecular mechanisms behind the control of Osx protein stability and suggests that targeting the Osx degradation pathway could enhance efficient osteogenesis and bone matrix regeneration. Additionally, the research reveals that the proteasome inhibitor USP17 plays a crucial role in regulating the stability of the Osx protein through deubiquitination, thereby adding a new dimension to our understanding of Osx regulation in the differentiation of osteoblasts.

## 4. Materials and Methods

### 4.1. Cell Culture and Differentiation

Human embryonic kidney (HEK 293) cells and mouse pre-myoblast cells (C2C12) were cultured in Dulbecco’s Modified Eagle Medium (DMEM; 12100046; Gibco™, Carlsbad, CA, USA) supplemented with 10% fetal bovine serum (FBS; S001-07; Welgene Inc., Daegu, Republic of Korea) and 1% antibiotic–antimycotics (#15240062; Gibco™) at 37 °C, 5% CO_2_. For C2C12 cell differentiation, fully confluent C2C12 cells were maintained in DMEM supplemented with 2% FBS and treated with 50 ng/mL BMP4.

### 4.2. Antibodies and Plasmids

Antibodies against Myc (9E10) and HA (12CA5) were acquired from Roche (Roche Applied Science, Basel, Switzerland). α-tubulin (B-5-1-2) and FLAG (F3165) were acquired from Sigma-Aldrich (Sigma, St. Louis, MO, USA). Runx2 (sc-390351) and Osx (sc-393325) were purchased from Santa Cruz (Santa Cruz Biotechnology, Santa Cruz, CA, USA). HA-Osx and Flag-Ub were constructed in a CMV promoter-derived expression vector (pCS4+), which contains either a HA-tag or Flag-tag. The Myc-tagged USP17 wild type and C89S(CS) mutant plasmids were generously provided by Prof. Kwang-Hyun Baek (CHA University).

### 4.3. Small Hairpin RNA (shRNA) and Transfection

For the knockdown of USP17, shRNA oligonucleotides were synthesized to target a 21-base pair (bp) sequence (GC AAA GAA AGA AAC CTC CTT A) in the mouse USP17 gene. The annealed oligonucleotides were ligated into the pSuper retro puro vectors (Oligoengine, Seattle, WA, USA). Overexpression and knockdown plasmids were transfected into HEK 293 or C2C12 cells using the polyethyleneimine (PEI; Polysciences, Warrington, PA, USA) transient transfection method.

### 4.4. Alkaline Phosphatase (ALP) Staining and ALP Activity Assay

For ALP staining, C2C12 cells were seeded in 24-well plates and allowed to differentiate for 72 h. Fully differentiated C2C12 cells were washed with phosphate-buffered saline (PBS) and fixed in 4% paraformaldehyde for 15 min at room temperature (RT). Then, the cells were stained using the BCIP/NBT Liquid Substrate System (B1911; Sigma-Aldrich) for 30 min at RT. Then, the ALP staining absorbance was measured at 480 nm. The ALP activity was measured using the SensoLyte^®^ pNPP Alkaline Phosphatase Assay Kit Colorimetric (1038; AnaSpec, Campus Drive, Fremont, CA, USA), according to the manufacturer’s instructions. The absorbance of ALP was measured at 405 nm.

### 4.5. Reverse Transcription-Quantitative Polymerase Chain Reaction (RT-qPCR)

C2C12 cells were seeded in 12-well plates and allowed to differentiate for 48 h. Total RNA was isolated from C2C12 cells using RNAiso Plus kit (TaKaRa, Tokyo, Japan) and reverse transcribed into cDNA using GoScript^TM^ Reverse Transcription System (Promega, Madison, WI, USA), according to the manufacturer’s instructions. RT-qPCR was performed using TB Green^®^ Premix Ex Taq™ (Tli RNaseH Plus) (TaKaRa) by a Bio-Rad real-time PCR system (CFX96, Bio-Rad, Hercules, CA, USA). Quantification of relative gene expressions was performed using GAPDH as an internal control. The following PCR primers were used: mALP forward 5′-ATC TTT GGT CTG GCT CCC ATG-3′ and mALP reverse 5′-TTT CCC GTT CAC CGT CCA C-3′; mOC forward 5′-GCA ATA AGG TAG TGA ACA GAC TCC-3′ and mOC reverse 5′-GTT TGT AGG CGG TCT TCA AGC-3′; mOsx forward 5′-TCG CAT CTG AAA GCC CAC TT-3′ and mOsx reverse 5′-CTC AAG TGG TCG CTT CTG GT-3; GAPDH forward 5′-AGG TCG GTG TGA ACG GAT TTG -3′ and GAPDH reverse 5′-GGG GTC GTT GAT GGC AAC A-3′.

### 4.6. Immunoblotting (IB) and Immunoprecipitation (IP)

Cells were lysed in lysis buffer [5 mM HEPES (pH 7.5), 150 mM NaCl, 1% NP-40, 0.25% sodium deoxycholate, 10% glycerol, 25 mM NaF, 1 mM EDTA, 1 mM Na_3_VO_4_, 250 μM PMSF, 10 μg/mL leupeptin, and 10 μg/mL aprotinin]. The whole-cell lysates were separated by sodium dodecyl sulfate–polyacrylamide gel electrophoresis (SDS–PAGE) and transferred to polyvinylidene fluoride (PVDF) membranes (Millipore, Billerica, MA, USA). The membranes were blocked in 5% skim milk, and then incubated in primary antibodies overnight at 4 °C. After washing with TBS–T [10 mM Tris–HCl (pH 7.4), 100 mM NaCl, and 0.1% (*v*/*v*) Tween 20], the membranes were incubated with horseradish peroxidase-conjugated (HPR) secondary antibodies for 1 h and visualized using Immobilon Western Chemiluminescent HRP Substrate (WBKLS0500, Millipore). Signals were detected and analyzed by the Amersham^TM^ ImageQuant^TM^ 800 system (GE Healthcare Life Sciences, Marlborough, MA, USA). For IP, HEK 293 cells were transfected with the indicated combinations of Myc-USP17, and HA-Osx. The whole cell lysates were collected by lysis buffer and incubated with appropriate antibodies and Protein A Sepharose CL-4B (#17096303; GE Healthcare Life Sciences). The protein–protein complex was eluted by heating at 100 °C, and then, detected by IB.

### 4.7. Glutathione S-Transferase (GST) Pulldown Assay

HEK 293 cells were transfected using HA-Osx expression plasmids. After 48 h, total cell extracts were lysed in an ice-cold lysis buffer. Aliquots of whole-cell lysates were incubated at 4 °C overnight and further incubated with glutathione–Sepharose beads for 2 h, which contained either GST or GST–USP17 protein. After washing twice with resuspension buffer [20 mM HEPES (pH 7.4), 120 mM NaCl, 10% glycerol, 2 mM EDTA], the bead–protein complex was eluted in SDS loading buffer, and then, detected by IB.

### 4.8. Cycloheximide Chase Analysis

HEK 293 cells were co-transfected with combinations of HA-Osx, Myc-USP17, and Myc-USP17-CS expression plasmids. After 24 h, cells were administered 40 μg/mL of cycloheximide (CHX, C1988; Sigma-Aldrich) for the indicated times (0 h, 2 h, 4 h, and 6 h). After CHX treatment, the cells were harvested and lysed in an ice-cold lysis buffer. Protein levels were analyzed by IB with tubulin used as the loading control.

### 4.9. Ubiquitination Assay

HEK293 cells were co-transfected with the indicated combinations of Flag-ubiquitin (Ub), HA-Osx, Myc-USP17, or Myc-USP17-CS expression plasmids. After 48 h, the cell lysates were prepared with lysis buffer. The same cell lysate amounts were incubated overnight with anti-HA, and then, incubated with Protein A Sepharose CL-4B for 2 h at 4 °C. The immunocomplexes were washed three times in lysis buffer, and then, analyzed by IB, while the Osx poly-ubiquitination status was analyzed using anti-Flag.

### 4.10. Statistical Analysis

All experiments were repeated at least three times. To determine differences between the two groups, Student’s *t*-test was performed, and differences between multiple groups were analyzed by one-way analysis of variance (ANOVA). A *p*-value of less than 0.05 was defined as being statistically significant.

## 5. Conclusions

In this study, we have elucidated the role of USP17 as a partner that promotes osteoblast differentiation by regulating the stability of the Osx protein, a crucial transcription factor involved in bone formation and osteoblast differentiation. USP17 appears to exert a positive regulatory role during osteoblast differentiation, likely through the stabilization of the Osx protein, thereby facilitating differentiation. This study introduces USP17 as a novel therapeutic target for bone disorders, such as osteoporosis, thus highlighting its specific function as a DUB (deubiquitinase) in the context of skeletal diseases.

## Data Availability

The data presented in this study are available in the article.
